# Interconnectedness of the Grinnellian and Eltonian Niche in Regional and Local Plant-Pollinator Communities

**DOI:** 10.3389/fpls.2019.01371

**Published:** 2019-11-06

**Authors:** Robert R. Junker, Martin H. Lechleitner, Jonas Kuppler, Lisa-Maria Ohler

**Affiliations:** Department of Biosciences, University of Salzburg, Salzburg, Austria

**Keywords:** biodiversity, floral traits, functional roles, impact, insect traits, networks, requirement, trait matching

## Abstract

Understanding the causes and consequences of coexistence and thus biodiversity is one of the most fundamental endeavors of ecology, which has been addressed by studying species’ requirements and impacts – conceptualized as their Grinnellian and Eltonian niches. However, different niche types have been mostly studied in isolation and thus potential covariation between them remains unknown. Here we quantified the realized Grinnellian niche (environmental requirements), the fundamental (morphological phenotype) and realized Eltonian niche (role in networks) of plant and pollinator taxa at a local and regional scale to investigate the interconnectedness of these niche types. We found a strong and scale-independent co-variation of niche types suggesting that taxa specialized in environmental factors are also specialized in their position in trait spaces and their role in bipartite networks. The integration of niche types thus will help to detect the true causes for species distributions, interaction networks, as well as the taxonomic and functional diversity of communities.

## Introduction

Understanding the mechanisms that underlie taxonomic and functional diversity and associated ecosystem functions is essential from a basic and applied perspective. Local and regional coexistence of taxa and thus biodiversity is promoted by niche partitioning and species interactions. Species of the same trophic level sharing the same habitat are predicted to differ in their environmental requirements enabling them to coexist (Abrams, 1983). Additional to environmental factors that represent important niche dimensions, interaction partners of other trophic levels can be regarded as a consumable resource and thus as further niche dimensions (Pauw, 2013). Therefore, interactions within and between trophic levels are key to the understanding of diversity patterns and associated ecosystem functions (Schleuning et al., 2015). Despite their indissociable effects on biodiversity, niche differentiation (within) and interaction networks (between trophic levels) have mostly been studied in isolation (Fründ et al., 2016; Anderson, 2017; Godoy et al., 2018; Gravel et al., 2018).

**Figure 1:**
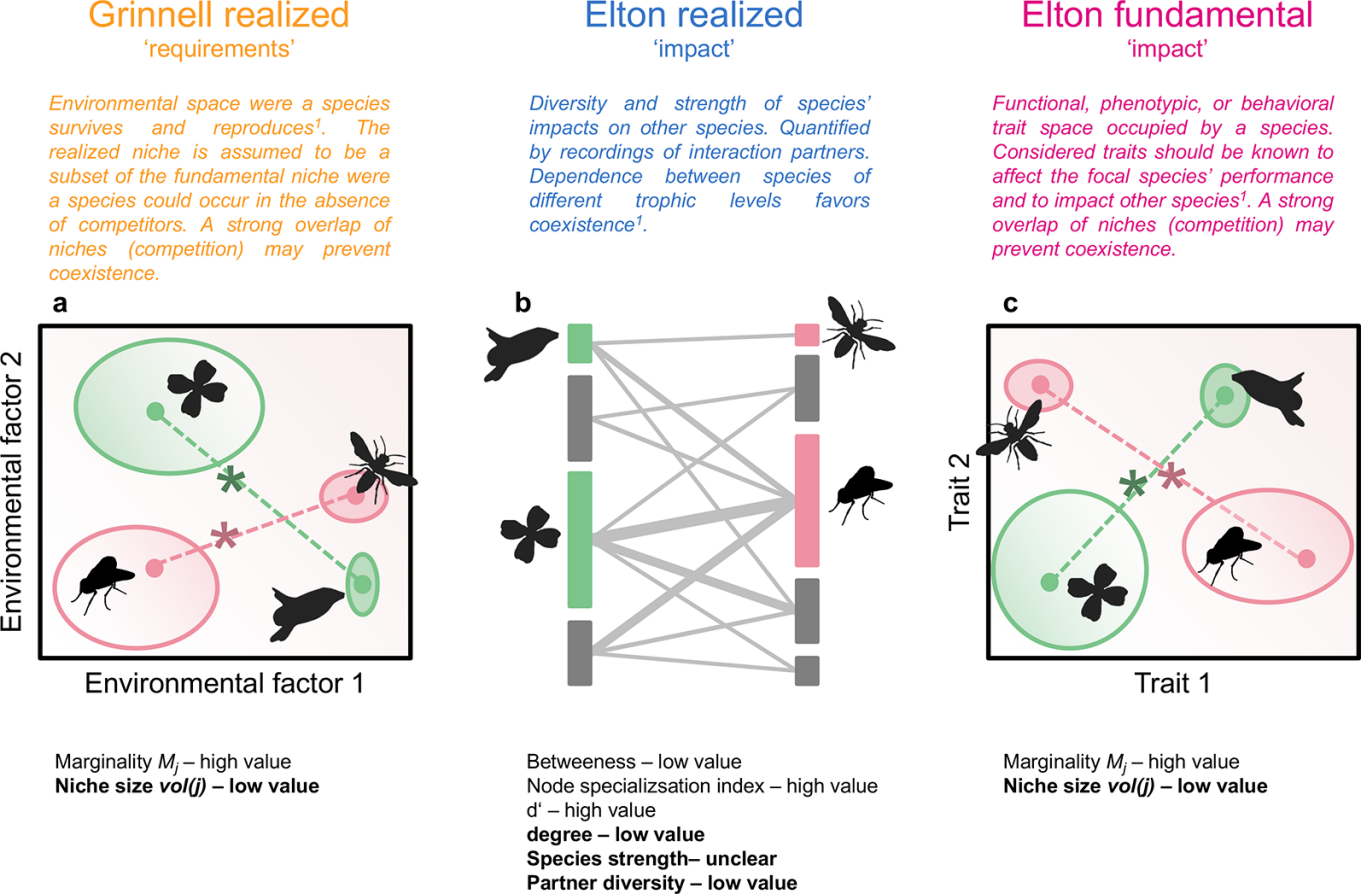
Hypothetical community of flowering plants and pollinators. Plant and animal species are characterized by their position and size in an environmental niche space (realized Grinnellian niche, **A**), by their role in a plant-pollinator interaction network (realized Eltonian niche, **B**), and their position and size in a trait space (fundamental Eltonian niche, **C**). Species positions in the niche spaces **(A**, **C)** are defined by the Euclidean distance (dashed lines) between the species’ centroid (closed circles, weighted mean position) and the centroid of all species of the same trophic levels (asterisks, note that only two species per trophic level are shown), i.e. their marginality *M* *_j_*. Species niche size, measured as niche volume *vol(j)* (Junker et al., 2016) is shown by the size of the open ellipses **(A**, **C)**, shading in ellipses denotes density within the occupied niche space. The approaches used in this study can be applied on datasets with an arbitrary number of dimensions. Species roles in interaction networks **(B)** are defined by betweenness, node specialization index, complementary specialization *d’*, degree, species strength, and partner diversity. Definitions are given in Dormann (2011). Vertical bars represent plant (left) or animal species (right), length of bars is proportional to number of interactions. Interactions between plants and animals are denoted by grey lines, width of lines is proportional to interaction frequency. Indices in bold are calculated independently from the other species in the same trophic level. The other indices inform about the niche positions/roles relative to the other species in the same trophic level. For each index, the value (low or high) for specialists is given. Results shown in **Figure 2** are roughly summarized in the panels **(A**-**C)**. ^1^ Devictor et al., 2010a.

Interactions between organisms within a trophic level (e.g. competition, facilitation) and across trophic levels (e.g. mutualism, antagonism) can be framed in the niche concept. In this context, the Grinnellian (“requirement”) and the Eltonian (“impact”) niche of organisms jointly shape the diversity and composition of communities (Chase and Leibold, 2003; Devictor et al., 2010a; **Figure 1**). Both types of niches are regarded as multidimensional constructs with dimensions defining the environment, resources, or phenotype of taxa (Junker et al., 2016). It is accepted that the multidimensionality of niches prevents the quantification of fundamental Grinnellian niches, because experimental tests are not feasible if multiple requirements are considered (Soberón and Arroyo-Peña, 2017). However, assessments of species’ distributions along environmental gradients allow a viable definition of their realized Grinnellian niches, i.e. the environmental requirements of species (McGill et al., 2006; Devictor et al., 2010a). The Eltonian niche, which considers the impact of species on their environment, can be measured either as the phenotype of species, representing the fundamental niche, or as their role in an interaction network, which can be defined as their realized niche in a given community (Devictor et al., 2010a; Cirtwill et al., 2018). Here, we integrate the Grinnellian and the Eltonian niche in order to obtain comprehensive insights into the interconnectedness of specialization/generalization of taxa in different niche types.

Multiple indices extracted from bipartite networks are available that inform about species specialization/generalization or the complementarity/redundancy of species within communities (Blüthgen et al., 2008; Cirtwill et al., 2018) and thus about the realized Eltonian niche of these taxa (Devictor et al., 2010a; **Figure 1**). The fundamental Eltonian niche and the realized Grinnellian niche can be positioned in Hutchinsonian *n*-dimensional hypervolumes (Hutchinson, 1957) that recently became directly quantifiable (Junker et al., 2016; Blonder, 2017). In *n*-dimensional hypervolumes, each taxon is characterized by *n* dimensions describing either its phenotype or environmental requirements. The hypervolume concept thus allows the quantification of the size of the niche (volume of the occupied space) as well as the position in the *n*-dimensional space (Junker et al., 2016; Kuppler et al., 2017; Junker and Larue-Kontic, 2018; **Figure 1**). The latter can be expressed as marginality, which is the distance of the species position to the mean position of all taxa in the species pool, and informs about the functional or environmental specialization (Mouillot et al., 2007; Devictor et al., 2010a; **Figure 1**).

The different types of niches consider different aspects of the ecology of taxa and the potential outcome of interactions with other community members of the same or other trophic levels (**Figure 1**). However, each niche type may also inform about the position of taxa in one of the other niche types emphasizing the interconnectedness of the requirements and impacts of taxa (Chase and Leibold, 2003; Kraft et al., 2015). By specifically testing the integrated nature of species requirements and impacts in communities it may be possible to identify causes for ecological specialization or generalization. For instance, a taxon that appears specialized in its requirements regarding environmental factors, may be in fact restricted in its environmental range due to the small Grinnellian niche of its obligate interaction partner. The interconnectedness of niche types has been shown in some studies: For instance, the environmental requirements of plants (Grinnellian niche) are closely related to the plants’ phenotype (fundamental Eltonian niche), which means that knowledge about environmental conditions is useful to predict the mean phenotype of plants in local plant communities and *vice versa* (Mouchet et al., 2010; Sundqvist et al., 2013; Junker and Larue-Kontic, 2018). Likewise, the role of taxa in interaction networks (realized Eltonian niche) has been shown to be influenced by the phenotype of the interaction partners (fundamental Eltonian niche). In consumer-resource relationships the difference in body size between interactions partners is predictive for interaction strength and network structure and thus community functionality (Brose et al., 2006; Otto et al., 2007). In plant-animal interactions, trait matching is a well known determinant of interactions: the beak size of birds matches well the size of fruits they consume (Dehling et al., 2014) and the proboscis length of flower visiting insect is correlated to the depth of nectar tubes (Stang et al., 2006). Accordingly, the position of the species in a trait space (fundamental Eltonian niche) has been shown to correspond to the role in interaction networks (realized Eltonian niche, Coux et al., 2016; Dehling et al., 2016). Apart from trait-matching, traits of resources can be viewed as requirements for foraging animals (Junker et al., 2013; Kuppler et al., 2017). As a consequence, resource traits predict the frequency of interactions, the role of taxa in networks, aggregate network indices, and biodiversity of communities (Junker et al., 2013; Junker et al., 2015; Olito and Fox, 2015; Dehling et al., 2016; Kuppler et al., 2017). In an empirical field study it is not feasible to consider all traits and requirements that affect the species’ ability to coexist. Therefore, estimates on the competition or interaction strength between taxa may vary if different traits and requirements (i.e. niche dimensions) are considered. However, we selected niche-dimensions that have been shown previously to be important determinants of species’ environmental requirements or interactions. Additionally, we quantified the impact niche of taxa – not their sensitivity niche, which is according to theoretical findings equally important for estimates of the probability to coexist (Meszéna et al., 2006) but impossible to quantify in a field study considering multiple taxa.

Environmental gradients are commonly regarded as natural experiments to study responses of species and communities to changing abiotic factors and to predict the fate of species and communities in times of global change (McGill et al., 2006; Sundqvist et al., 2013). Both species and community properties change along gradients: communities vary in taxonomic, phylogenetic, and functional diversity and composition (Cornwell and Ackerly, 2009; Devictor et al., 2010b; Junker and Larue-Kontic, 2018) and species vary in traits due to local adaptation or phenotypic plasticity (Körner, 2003; Sundqvist et al., 2013; Merilä and Hendry, 2014). Responses of species, communities, and interaction networks to environmental gradients can be observed across gradients ranging from the global scale (Schleuning et al., 2012; Lamanna et al., 2014) to gradients in geographically restricted areas (Benadi et al., 2014; Junker and Larue-Kontic, 2018). Additionally, microclimatic heterogeneity, which is variation in abiotic factors within small and clearly defined areas, has recently been shown to affect the spatial distribution of plant species as well as the functional composition of sub-local communities (Scherrer and Körner, 2011; Opedal et al., 2015; Blonder et al., 2018). Ecological properties and processes are thus affected by different spatial scales and the niches of taxa may vary in a scale-dependent manner (Devictor et al., 2010a).

In this study, we specifically tested the interconnectedness of the Grinellian and Eltonian niche of taxa at a regional and local scale in order to relate species specialization/generalization in different niche types. We recorded plant-pollinator interactions along an elevational and a microclimatic gradient. Additionally, we recorded morphological traits of flowers and flower-visiting insects as well as their requirements on temperature and aspect of the habitat. Using these data, we tested the following hypotheses: 1.) the degree of specialization/generalization of organisms in one niche type correlates to the degree of specialization/generalization in other niche types. 2.) The niches of organisms as well as the interconnectedness of the niche types is independent on the spatial scale. 3.) Animals and plants are more likely to interact with each other if they share the same realized Grinnellian niche (environmental requirements). By considering different niche types of taxa at different spatial scales, we are aiming at generating a multifaceted understanding of the mechanisms underlying coexistence and spatial biodiversity patterns.

## Material and Methods

### Study Sites

At the regional scale we studied communities at eight elevations between 1,227 m a.s.l. and 2,636 m a.s.l. close to the National Park Hohe Tauern in the Austrian Alps between May and September in 2016 and 2017. Sites were selected based on accessibility and to evenly cover the elevational gradient. In each site we established three transects (3 x 30 m) that were visited once a month for several days throughout the vegetation period. Per transect two 2 x 2 m plots were established for monthly vegetation surveys (*n* = 121) following a modified abundance-dominance scale based on Braun-Blanquet (1964) to assess the abundance (cover per species in percent) of the present plant species. For statistical analysis we used the recorded values of plant cover in percent and substituted *+* and *r* with 0.5 and 0.1, respectively. Flower-visitor interactions were recorded for at least three days per monthly visit. At both ends of each transect one temperature logger (DS1921G-F5 Thermochron iButtons, Maxime Integrated Products, Sunnyvale, CA, USA) was buried (3 cm in depth) during the vegetation period, recording soil temperature continuously every 30 min from May to September. Additionally, we assessed the aspect of each transect using the software QGIS (QGIS Development Team, 2009). North was set to 0°, south to 180°, east and west to 90°.

At the local scale, we established 30 plots (1.5 x 1.5 m) within a hectare at a meadow at 2,273 m a.s.l. that were studied between May and September 2016. Sites were selected to represent the variability in aspect and slope within the meadow. Vegetation surveys after Braun-Blanquet were carried out as described above once per plot during the vegetation peak. On each plot, we recorded flower-visitor interactions at four days throughout the field season for a period of 5 min each (i.e. 20 min observation time per plot), following a randomized order. Soil temperature and aspect was assessed as described above.

Location, elevation, number of plant and insect species, temperature, and aspect of transects (regional scale) and plots (local scale) is given in **Supporting Information 1**.

### Flower-Visitor Interactions

On sunny days we recorded flower-visitor interactions between 8:45 and 18:00 h. In total, we conducted 76 observation days and 289 h of observation time. Interactions were recorded by up to four persons simultaneously. Each transect plot was sampled by slowly walking along transects and catching all insect encountered while contacting a flower (Junker et al., 2013). Also plant species with long nectar tubes or concealed floral parts were checked for insects inside these tubes. All arthropods were collected, labeled, and stored in a freezer until further treatments.

### Flower and Insect Morphology

For each flowering entomophilous plant species encountered at transects or plots, we measured the depth and width of the nectar holder tube and the position of the anthers relative to the floral surface using a caliper rule in *n* = 7–10 individuals per species and elevation. Nectar width was defined as the diameter of the nectar holder tube, which limits access by a visitor. The nectar depth was defined as the distance between the constricting part of the flower to the bottom of the flower. For open or bowl-shaped flowers the nectar width was defined as the flower diameter and nectar depth was set to zero. Anther position was measured along the same axis as the nectar depth and defined as the distance between the anthers and the constricting part of the flower, which means that positive values indicate anthers above the floral surface and negative values indicate that anthers are hidden in nectar tubes.

For each insect taxon we measured the length of the extended proboscis, the width of the head capsule, and the total body length using a caliper rule in *n* = 7–10 individuals per species and elevation. The body length was defined as the distance between the head and the tip of the abdomen, not including body appendages like antennae, genitalia or cerci. Additionally, we assigned the number of pollen grains attached to four body regions (head, thorax, abdomen, legs) to one of the following categories: 0 (no pollen grains), 1 (1–4 pollen grains), 2 (5–10 pollen grains), 3 (11–100 pollen grains), 4 (> 100 pollen grains). We included only those insect taxa that potentially act as pollinators, i.e. those that received an average of category > 1 over all body regions and individuals sampled in the further analysis. Insects were identified by experts or based on literature. Morphological measurements were performed at the species level. Most species, however, occurred in very low numbers preventing a realistic estimation their niches. Therefore, for the statistical analysis we considered the family level (in rare cases higher taxonomic levels containing few individuals if identification was not possible) in order to increase sample size per taxon. For each taxon (family, order), we pooled all species in this taxon to estimate niches. A full list of species is given in supporting information 2. Mean trait values of plants and animals are given in **Supporting Information 3** and **4**.

### Definition of the Realized Grinnellian Niche

The realized Grinnellian niche of plants and animals (i.e. their environmental requirements) was defined as the mean seasonal temperature and aspect of transects or plots where the taxa were recorded using abundance as weight. Mean seasonal temperature per plot is the mean of all recordings of the temperature logger located at each plot. Prior to analysis data were standardized between 0 and 1 [x’ = (x-min)/(max-min)]. The realized Grinnellian niche of taxa was calculated separately for the regional (transects) and local pool (plots) of taxa. Plant abundance *a* *_pj_* per transect/plot was assessed based on the coverage estimated in vegetation analyses (mean proportional foliage cover during the field season), animal abundance *a* *_pj_* as number of interactions observed per transect/plot. Thus, we defined the environmental space of taxa as the two-dimensional area occupied by each taxon *j* based on the weighted mean seasonal temperature and aspect of transects or plots *p* where *j* was encountered, weighted by the abundance of *j* on *p*. As abundance *a* *_pj_* of plant species we multiplied the percentage of the cover (often < 1%) with 100 and rounded the value to the nearest integer, which is required for further analysis. Each temperature and aspect value of the transects or plots the taxa where recorded on was *a* *_pj_* times added to a vector defining the realized Grinnellian niche of plant and animal taxa *j*. Accordingly, for each taxon *j* we obtained two vectors containing temperature or aspect values with the length $V=\sum_{p=1}^{P}a_{pj}$ with *P* being the total number of transects or plots a taxon was recorded on. Thus, the two-dimensional area occupied by taxa *j* was expressed as a matrix with *V* rows and two columns containing temperature or aspect values. Using this matrix, we calculated the abundance weighted mean position *J* of each taxon *j* and the centroid *c* of all plant or animal taxa (separately) in the two-dimensional area. The Euclidean distance between *J* and *c* was defined as the marginality *M* *_j_* of taxon *j*, which is the deviation of taxon *j* from the mean taxon of the same trophic level. Additional to marginality *M* *_j_*, we calculated the niche size *vol(j)* of each taxon *j* by using standard settings in function *dynRB_VPa(data)* and aggregation method *mean* implemented in the R package *dynRB* (Junker et al., 2016).

### Definition of the Fundamental Eltonian Niche

The fundamental Eltonian niche of plants and animals (i.e. their potential impact on other organisms in the community) was defined based on the plants’ and animals’ phenotype. For each plant species we phenotyped *n* = 7 or 10 individuals (depending on the abundance) characterizing the fundamental impact niche of the plant species. For each animal taxon we used the phenotypes of the species (*n* = 10 individuals per species) pooled in the family as impact niche. Marginality *M* *_j_* and niche size *vol(j)* of taxon *j* were calculated as described above and also separately for the regional and local pool of taxa.

### Definition of the Realized Eltonian Niche

The realized Eltonian niche of plants and animals (i.e. their impact on other organisms in the community) was defined as their role in a bipartite network summarizing the interactions between plants and animals at all transects (regional) or plots (local species pool). Based on the interactions recorded in the field we compiled a matrix with animal taxa as rows and plant species as columns and the number of interactions between plants and animals as entries in the cells. Based on these matrices for the regional and local species pool we calculated the degree (number of interaction partners), species strength (dependence of the organisms of one trophic level on a species of the other trophic level), betweenness (fraction of all shortest paths in a one-mode network that involve the focal species as node), partner diversity (Shannon diversity of the interaction partners), node specialization index (the mean path length in a one-mode network between the focal species and all other species of the same trophic level), and complementarity specialization *d’* (exclusiveness of interactions partners of the other trophic level) for each plant and animal taxon using the function *specieslevel(data)* implemented in the R package *bipartite* (Dormann, 2011). A detailed description of the indices describing taxon-specific roles in bipartite networks can be found in Dormann (Dormann, 2011).

### Interconnectedness of Niche Types

In order to test whether the realized Grinellian and the fundamental Eltonian niche defined as marginality *M* *_j_* and niche size *vol(j)* are related to the taxon-specific roles (realized Eltonian niche), we performed Pearson’s product-moment correlations between all pairs of the 10 indices. Correlation analyses were performed separately for animals and plants and the datasets based on the local and the regional species pool. Based on the correlation matrices we created weighted networks visualizing the correlations between the indices with *r* *^2^* as weight (only significant correlations were considered) using the R package *igraph* (Csardi and Nepusz, 2006). Next, we calculated the modularity of the correlation networks and recorded the affiliation of each index to one of the modules using the R package *igraph*. Additionally, we calculated the integration of the correlation matrices. Here, integration informs about the strength of the overall co-variation between the indices. We followed a standard method that corrects for varying sample sizes (Wagner, 1984; Herrera et al., 2002; Junker et al., 2018). For each correlation matrix we calculated the variance of the eigenvalues, which is the integration index and standardized it for varying samples size allowing comparison of the integration values across matrices despite varying samples sizes.

To test whether plants and animals occupy the same niches at the regional and local species pool, we correlated the 10 indices defining niche positions and roles received by the plants and animals that occurred in both data sets. Additionally, we tested whether the interaction frequency of plants and animals affected indices defining niche position and role.

### Positioning of Interactions in the Realized Grinnellian Niche Space

We tested whether plant and animal taxa that are similar in their realized Grinnellian niche are more likely to interact with each other and to interact in higher frequencies than plants and animals that are positioned further apart in the realized Grinnellian niche space. Therefore, we quantified the “*weighted mean realized edge length* 〈*E* *_r_*〉“ in the regional and local network and compared it to the “*weighted mean expected edge length* 〈*E* *_e_*〉“ assuming random interactions between plants and animals. The realized edge length *E* *_r_* of the interaction between plant *j* and animal *i* is defined as the Euclidean distance between the plant’s position *J* in the niche space with temperature and aspect as dimensions and the animal’s position *I* in the same space. The weighted mean of all *E* *_r_* in a network with interaction frequency as weight is defined as “*weighted mean realized edge length* 〈*E* *_r_*〉“. To test whether 〈*E* *_r_*〉 deviates from a null model expectation, we generated *n* = 10,000 random two-way tables with fixed row and column sums using Patefield’s algorithm (Patefield, 1981) and calculated the “*weighted mean expected edge length* 〈*E* *_e_*〉“ for each of the simulations as described above. A significant deviation of 〈*E* *_r_*〉 from 〈*E* *_e_*〉 is indicated if < 5% of all 〈*E* *_e_*〉-values are lower than 〈*E* *_r_*〉.

## Results

Overall we recorded *n* = 10,405 interactions (*n* = 9,390 at the regional scale, *n* = 1,015 at the local scale). After removal of insect families that did not carry pollen in sufficient numbers (i.e. mean pollen category ≤ 1), a total number of *n* = 6,896 (*n* = 6,194 and *n* = 702) interactions was considered for further analysis. We considered a total of *n* = 130 plant species and *n* = 60 animal taxa for further analysis. All of the taxa were phenotyped.

**Figure 2:**
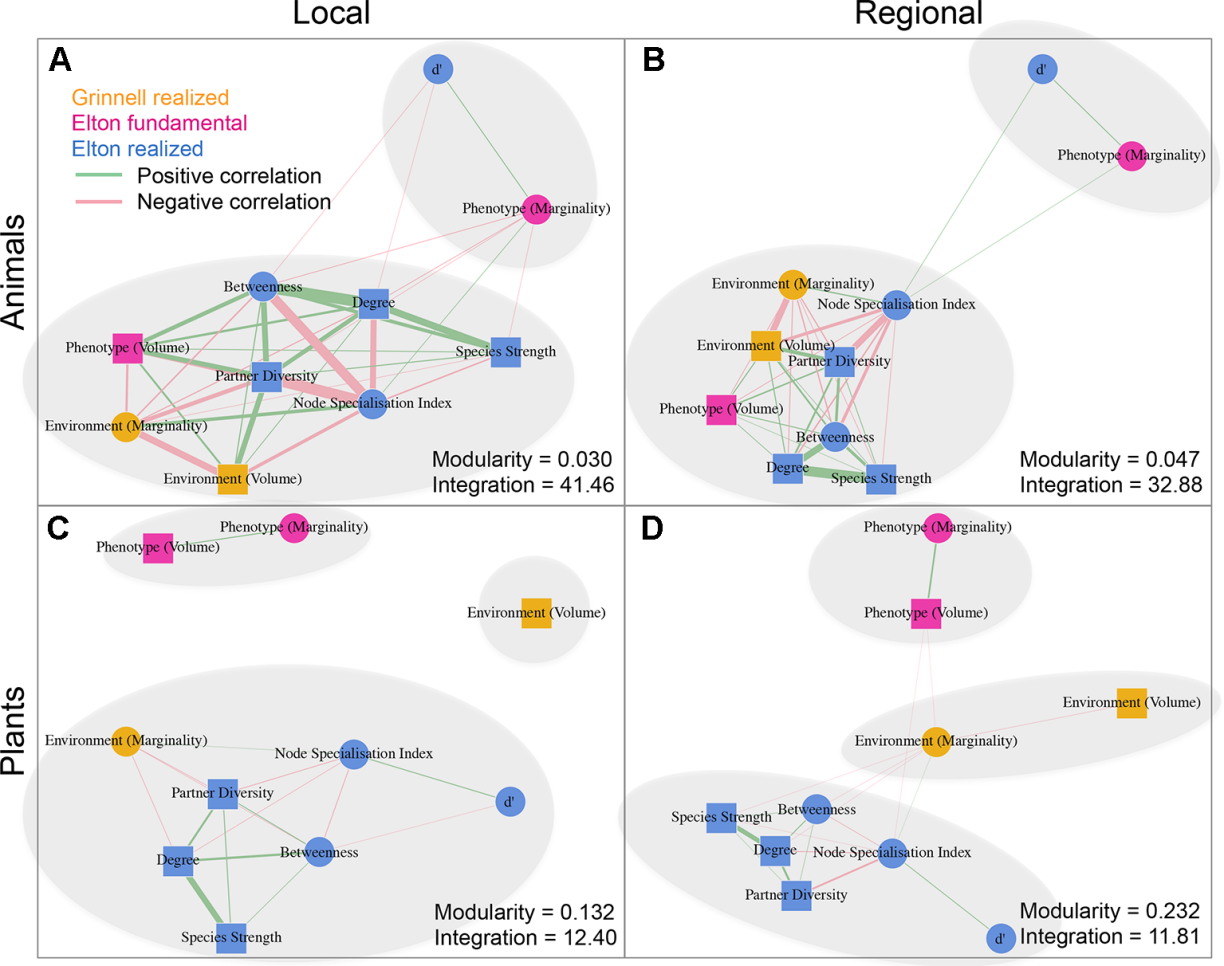
Networks based on correlation matrices between indices informing about the position/role of taxa in the realized Grinnellian niche (orange, marginality *M* *_j_* and niche size *vol(j)* based on the temperature and aspects of the taxa’s habitat), the fundamental Eltonian niche (pink, marginality *M* *_j_*, and niche size *vol(j)* based on the taxa’s morphology), and the realized Eltonian niche (blue, degree, species strength, betweenness, partner diversity, node specialization index, and complementarity specialization *d’* calculated from bipartite networks). Datasets on animals **(A**, **B)** and plants **(C**, **D)** as well as considering the local **(A**, **C)** and regional **(C**, **D)** species pool are shown separately. Squares represent indices that are calculated for each taxon independently from the taxa of the same trophic level. Circles represent indices that are calculated in dependence of the other community members of the same trophic level. Positive and significant correlations are shown as green edges, negative and significant correlations as red edges. Width of edges is proportional to *r* *^2^*. Note that specialists receive high values in some indices and low values in others (see **Figure 1**). Therefore, both negative and positive correlation may indicate that species that are specialized in one index are also specialized in another index, depending on the pair of indices under consideration. Usually, significant correlations indicated that taxa specialized on one niche type were also specialized in another niche type. The exception from that rule is the marginality *M* *_j_* and niche size *vol(j)* of the fundamental Eltonian niche that was positively correlated in plant species indicating that species that are more similar to the average plant (low *M* *_j_*) and thus are more generalized in their niche position are phenotypically more specialized, i.e. have small niche volumes. Modules are shown by grey ellipses. Modularity and integration are shown for each data set informing about the strength of co-variation between indices. Networks analysis and calculation of modularity have been performed using the R package *igraph* (Csardi and Nepusz, 2006). Results are roughly summarized in **Figure 1**.

We detected a number of significant correlations between the indices informing about the position/role of taxa in the realized Grinnellian niche (marginality *M* *_j_* and niche size *vol(j)* based on the temperature and aspects of the taxa’s habitat), the fundamental Eltonian niche (marginality *M* *_j_* and niche size *vol(j)* based on the taxa’s morphology), and the realized Eltonian niche (network position: degree, species strength, betweenness, partner diversity, node specialization index, and complementarity specialization *d’*, **Figure 2**). Note that specialists receive high values in some indices and low values in others (see **Figure 1**). Therefore, both negative and positive correlation may indicate that species that are specialized in one index are also specialized in another index, depending on the pair of indices under consideration. Usually, significant correlations indicated that taxa specialized on one niche type were also specialized in another niche type (**Figure 2**). The exception from that rule is the marginality *M* *_j_* and niche size *vol(j)* of the fundamental Eltonian niche that was positively correlated in plant species indicating that species that are more similar to the average plant (low *M* *_j_*) and thus are more generalized in their niche position are phenotypically more specialized, i.e. have small niche volumes. Integration values (i.e. overall co-variation) based on correlations of indices was more pronounced at the local scale than on the regional scale and more pronounced in animal than in plant communities (**Figure 2**). Likewise, modularity of networks was lowest in the dataset on the local scale considering animals and highest in the dataset on the regional scale considering plants (**Figure 2**). Networks visualizing co-variation of the animals’ positions/roles in the niche types featured two modules and high integration (**Figures 2A, B**). Most indices were assigned to one module, except for marginality *M* *_j_* in the fundamental Eltonian trait space and complementarity specialization *d’* (realized Eltonian niche) that were assigned to another module (**Figures 2A, B**). Networks visualizing co-variation of the plants’ positions/roles in the niche types featured three modules and low integration (**Figures 2C, D**). In these cases, niche types formed their own modules with weak (but significant) correlations to indices in other modules (**Figure 2**), except for the marginality *M* *_j_* in the realized Grinnellian niche that was assigned to the same module as the indices informing about the realized Eltonian niche in the local dataset (**Figure 2**). Results are roughly summarized in **Figure 1**. Animal taxa occupied mostly the same position and mostly had the same role in the network on the regional and the local scale, i.e. seven out of the 10 indices considered significantly correlated (**Table 1**). In plant species, local and regional positions/roles did mostly not correspond to each other, i.e. 7 out of the 10 indices considered did not significantly correlate (**Table 1**). The number of interactions observed for plants and animals mostly correlated to the taxa’s roles in network (realized Eltonian niche) except for *d’* (**Table 2**). The indices informing about the realized Grinellian and the fundamental Eltonian niche were mostly independent of the number of interactions, except for the marginality *M* *_j_* in the realized Grinnellian nice that was negatively correlated to number of interactions in most cases (**Table 2**). Variation in environmental factors and morphological traits was mostly smaller within taxa than between taxa (see **Supporting Information 5**).

**Table 1:** Correlation of the position/role of taxa quantified using the datasets of the local and regional scale.

Index	Animals				Plants			
	*t*	*df*	*p*	*r^2^*	*t*	*df*	*p*	*r^2^*
Phenotype (marginality)	1.880	15	0.0797	0.191	11.823	21	**<0.001**	0.869
Phenotype (volume)	1.904	15	0.0763	0.195	1.806	21	0.0853	0.134
Environment (marginality)	4.296	15	**0.0006**	0.552	0.461	17	0.6504	0.012
Environment (volume)	4.065	15	**0.0010**	0.524	1.954	18	0.0665	0.175
Degree	6.760	15	**<0.001**	0.753	3.697	21	**0.0013**	0.394
Species strength	6.067	15	**<0.001**	0.710	2.328	21	**0.0300**	0.205
Node specialization index	2.927	15	**0.0104**	0.364	0.212	21	0.8342	0.002
Betweenness	5.748	15	**<0.001**	0.688	0.595	21	0.5582	0.017
Partner diversity	2.800	15	**0.0135**	0.343	1.738	21	0.0969	0.126
*d’*	1.568	15	0.1377	0.141	0.855	21	0.4020	0.034

Significant Pearson’s product-moment correlations indicate that taxa occupy the same position or have the same role in the local and the regional community. Significant correlations are highlighted in bold.

**Table 2:** Correlation of the total number of interactions of plants and animals with their position/role quantified using the datasets of the local and regional scale.

	Animals		Plants	
	Local	Regional	Local	Regional
Phenotype (marginality)	−0.478	−0.324	0.051	−0.126
Phenotype (volume)	**0.536**	0.337	0.287	0.080
Environment (marginality)	−0.450	−**0.504**	−**0.538**	−**0.341**
Environment (volume)	0.372	**0.457**	0.122	**0.260**
Betweenness	**0.852**	**0.872**	**0.788**	**0.607**
Degree	**0.990**	**0.934**	**0.620**	**0.771**
Species strength	−**0.635**	−**0.493**	−0.382	−**0.283**
Partner diversity	**0.765**	**0.703**	**0.685**	**0.442**
Node specialization index	**0.571**	**0.468**	0.350	**0.243**
d’	−0.413	−0.231	−0.287	−0.091

Significant correlations suggest that the number of interactions observed for a plant or animal taxon is predictive for the position or role of the taxon in the community. Shown are r-values of Pearson’s product-moment correlation. Significant correlations are highlighted in bold. Note the comparable patterns in plants and animals.

**Figure 3:**
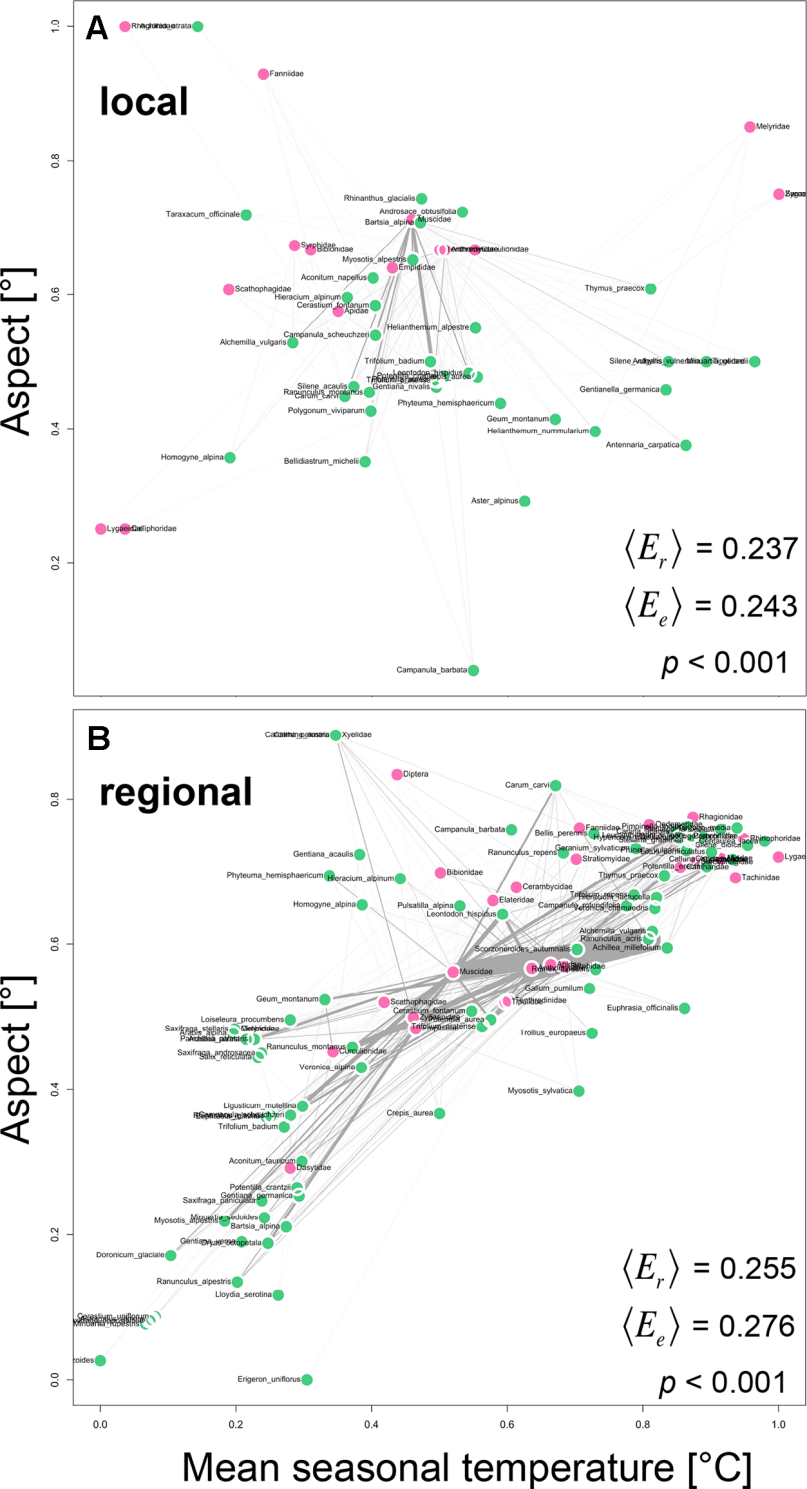
Positioning of plant-animal interactions into the realized Grinnellian niche space based on the mean seasonal soil temperature [°C] and the aspect [°] of the transects/plots were the interactions occurred, weighted by abundance. Prior to analysis data were standardized between 0 and 1 [x’ = (x-min)/(max-min)]. Thus, coordinates are unitless. Local **(A)** and regional **(B)** networks are shown. Each circle represents one plant species (green) and one animal family (pink) and is located at the abundance weighted mean position *J* of each taxon *j* in the two-dimensional niche space. Interactions are shown as gray edges between plants and animals. Edge width is proportional to interaction strength, i.e. the number of interactions observed between a plant and an animal taxon. Based on the edges, the *weighted mean realized edge length* 〈*E* *_r_*〉 was calculated with number of interactions as weight. To test whether 〈*E* *_r_*〉 deviates from a null model expectation, we generated *n* = 10,000 random two-way tables with fixed row and column sums using Patefield’s algorithm (2011) and calculated the *weighted mean expected edge length* 〈*E* *_e_*〉 for each of the simulations as described above. A significant deviation of 〈*E* *_r_*〉 from 〈*E* *_e_*〉 is indicated if < 5% of all 〈*E* *_e_*〉-values are lower than or equal to 〈*E* *_r_*〉.

Plant and animal taxa that were similar in their realized Grinnellian niche were more likely to interact with each other and to interact in higher frequencies than plants and animals that are positioned further apart in the realized Grinnellian niche space (**Figure 3**). Accordingly the “*weighted mean realized edge length* 〈*E* *_r_*〉“ was significantly shorter (local: 0.185, regional: 0.255) than the “*weighted mean expected edge length* 〈*E* *_e_*〉“ (local: 0.194, regional: 0.276) in both the local (**Figure 3A**, *p* < 0.001) and the regional network (**Figure 3B**, *p* < 0.001).

## Discussion

Environmental requirements (realized Grinnellian niche), the phenotype of organisms (fundamental Eltonian niche) and the organisms’ interactions with taxa from other tophic levels (realized Eltonian niche) are mostly studied in isolation (Fründ et al., 2016; Anderson, 2017; Godoy et al., 2018; Gravel et al., 2018). Each of these niche types informs about the specialization/generalization of organisms regarding differing aspects of their ecology. For a full assessment of the causes and consequences of specialization/generalization a comprehensive view on all of the niche types may be helpful. We found that niche types are strongly interconnected in animals, whereas covariation in plant species’ positions and roles in niches was less pronounced. These results indicate that animal taxa that are specialized in one niche type are also specialized in the other niche types. That means that animal taxa that have a narrow environmental niche (small niche size, realized Grinellian niche), also occupy a small trait space (fundamental Eltonian niche), and are specialized regarding interaction partners (realized Eltonian niche). Environmental marginality, i.e. the deviation of an animal’s niche position from the mean niche position of all other animals, was negatively correlated to the environmental niche size, meaning that animals specialized in the niche position also had a smaller environmental niche. Phenotypic marginality varied independently of the environmental niche and the size of the phenotypic niche, but was associated with the complementary specialization *d’*. This correlation indicates that animals with morphologies that differ from most of the other animals also occupy a unique niche in the realized Eltonian niche, i.e. interact with plants that are not or seldom visited by other animals. In plants, environmental marginality was associated with plant species’ role in interaction networks. Otherwise, the realized Grinellian niche, as well as the fundamental and realized Eltonian niches largely varied independently from each other. Earlier findings also suggested a stronger link between the fundamental and realized Eltonian niche in animals than in plants (Coux et al., 2016). Interestingly, plants with larger phenotypic niche sizes deviated more strongly from the mean phenotype of all plant species (marginality) than plant species with smaller phenotypic niche sizes. Additionally, to the interconnectedness between the niche types of organisms within a trophic level, our analysis also revealed that the similarity in the realized Grinellian niche of plants and animals is positively associated with likelihood and the frequency of interactions between the tropic levels (realized Eltonian niche).

Our results demonstrate the interconnectedness of different types of niches, particularly in animals. The correlation between the fundamental and the realized Eltonian niche may be a signal of trait matching: animals with morphologies that differ from the mean morphology across all taxa in the community (and thus also from the majority of the other taxa) are likely to find fewer and/or only a specific set of partners in the other trophic level (Junker et al., 2013; Kaiser-Bunbury et al., 2014), resulting in a higher specialization in bipartite networks. In contrast, animals with a high within taxon variation may be able to exploit a larger set of flowering plant species and thus appear generalized in the network. Note that we lumped species of the same family into one taxon in our analysis, which may increase the intra-taxon variation. However, variation within taxa in environmental factors and morphological traits was usually smaller than variation between taxa in our data and it has been shown that interaction networks considering insect family level have highly similar properties as networks on the species level (Trojelsgaard et al., 2015). Additionally, number of interactions (typically in taxa including more species) showed no correlation to most indices characterizing the realized Grinnellian and the fundamental Eltonian niche.

The positive correlation between the two measures of the fundamental Eltonian niche in plants suggests that species with specialized position (high marginality) also feature a higher intraspecific variability (niche volume). Species with phenotypes close to the mean of all species may experience stronger competition for pollinators than species with strongly deviating phenotypes, which may lead to a reduced intraspecific variability in the former group according to niche packing theory (Violle et al., 2012).

The correlation between the realized Grinnellian niche and the realized Eltonian niche suggests that spatially restricted taxa, e.g. those that have specific requirements on their environment, have access to only a limited set of interactions partners that are able to grow under the same environmental conditions (Devoto et al., 2005). Alternatively, a small realized Grinnellian niche of obligate interaction partner(s) may force specialized pollinators into the same realized Grinnellian niche. Interestingly, we found the same pattern at regional and the local scale although niche partitioning based on environmental factors should be less pronounced at the local scale (McGill, 2010) confirming the importance of microclimatic heterogeneity in explaining diversity and interaction patterns (Blonder et al., 2018).

The explanation for a correlation between the realized Grinnellian niche and the fundamental Eltonian niche may be twofold: First, phenotypic plasticity and local adaptation, both increasing intraspecific variation, allow species to adapt to different environments (Gonzalo-Turpin and Hazard, 2009) and thus a large fundamental Eltonian niche (high intraspecific variation) may allow species to establish in varying environments leading to a large realized Grinnellian niche. Second, the smaller intraspecific variation of species that inhabit extreme environments, i.e. those that deviate from the mean habitat (negative correlation between the volume of the fundamental Eltonian niche and the marginality of the realized Grinnellian niche) supports the notion that environmental filtering becomes more important in extreme environments (Chase, 2007; Hoiss et al., 2012).

Plant and animal taxa that meet in space, i.e. those that have similar realized Grinnellian niches, were more likely to interact in high frequencies than taxa that differed more strongly in their environmental requirements, which has been suggested by studies considering a regional scale (Olesen and Jordano, 2002), but not on the local scale. Thus, this finding again reemphasizes the consideration of microclimatic heterogeneity in ecological studies (Opedal et al., 2015). Furthermore, it shows that the realized Grinnellian niche of taxa has influence on the microstructure of networks and thus their role in networks.

The covariation between the indices describing the taxa’s position or role in niche types was nearly identical in the datasets sampled along the elevational gradient comprising 1,409 m in altitude and sampled within a single alpine meadow suggesting a scale-independent interconnectedness of the niche types. Likewise, the position or role in niche types of individual taxa often was the same at the local and the regional scale, i.e. taxa often received similar relative values in the indices in both datasets, also suggesting a scale-independent position of taxa in their communities. The lack of significant correlations between plant species’ positions or roles at the regional and local scale in 7 out of 10 indices suggests that their niches are more affected by the composition of the community than the animals’ niches. The immobility of plants may increase the importance of stochastic processes such as dispersal and it may decrease the plants’ ability to quickly adapt or respond to small-scale differences in environmental factors or large-scale shifts in temperature at times of climate warming. In contrast, mobile insects are able to find their preferred sites within a season and therefore may have more stable niches at different scales. Thus, insect communities may have the ability to flexibly assemble according to the environmental conditions leading to more invariant communities and thus more similar niche positions across scales.

The interconnectedness of niche types detected in our study emphasizes the integrated nature of species requirements and impacts in communities and gives rise to questions regarding the causes for ecological specialization or generalization. Therefore, the causes for specialization in one of the niche types may lie in the specialization in another niche type and knowledge about the interconnectedness of niches may thus facilitate the identification of limiting factors for organisms. Future work may expand our approach to other systems such as plant-herbivory or host-parasite interactions. We conclude that the integration of niche types may help to detect the true causes for species distributions, interaction networks, as well as the taxonomic and functional diversity of communities. Accordingly, conservation efforts may benefit from holistically characterize the niche of organisms.

## Author Contributions

RJ conceived the study. RJ, ML, JK, and LO designed the study. ML, JK, and LO conducted fieldwork. RJ and ML performed statistical analyses. RJ drafted the manuscript and all authors contributed to the final version.

## Funding

The study was funded by the Austrian Science Fund (FWF, P29142-B29).

## Conflict of Interest

The authors declare that the research was conducted in the absence of any commercial or financial relationships that could be construed as a potential conflict of interest.
